# Measuring Sports’ Perceived Benefits and Aggression-Related Risks: Karate vs. Football

**DOI:** 10.3389/fpsyg.2020.625219

**Published:** 2021-01-18

**Authors:** Teresa Limpo, Sid Tadrist

**Affiliations:** ^1^Faculty of Psychology and Education Sciences, University of Porto, Porto, Portugal; ^2^KWF Dynamic Karate, London, United Kingdom

**Keywords:** karate, football, perceived aggressiveness risks, perceived benefits, sports

## Abstract

Little is known about people’s perceived benefits and risks of sports, despite their role in shaping people’s intentions to engage in them. Here, we developed and tested a scale to measure perceived physical, emotional, cognitive, and social benefits as well as aggression-related risks of karate and football. Additionally, we compared these perceptions within and between these two sports, as well as among undergraduates with current/former participation in different types of physical activity (viz., martial artists, team sports players, participants in other types of physical activity, and non-participants). After a literature review, we created a 5-factor scale with 20 items administered to 184 undergraduates, along with questions about physical activity participation. After removing five items, confirmatory factor analyses supported the factor structure of the scale. Factor loadings and reliability indices were acceptable, though less than desirable results were found concerning the average variance extracted of all benefits dimensions and the reliability of the social benefits dimension. Analyses of variance showed that: (a) physical benefits were seen as the salient outcomes of karate and football, though martial artists perceived karate’s physical, emotional, and social benefits to the same extent; (b) in comparison to football, karate was perceived to bring more emotional and cognitive benefits and to entail less aggressiveness risks; (c) karate and football perceptions varied as a function of participant’s involvement in physical activity. This study presents a promising instrument to gather information on people’s perceptions about karate and football, which can be used to foster people’s engagement in them.

## Introduction

The benefits of physical activity (PA) – defined as “any bodily movement produced by skeletal muscles that results in energy expenditure” (Caspersen et al., 1985, p. 128), such as sports, conditioning activities, walking, active recreation, or play – are well established (U.S. Department of Health and Human Services, 2018). Evidence concerning the specific benefits of different sports is also growing (Bu et al., 2010; Oja et al., 2015). However, less is known about the degree to which people perceive those benefits. This was the goal of the present study, which compared undergraduates perceived benefits and aggressiveness risks in football vs. karate.

Based on a thorough literature review, the report “Designed to Move” – presented by American College of Sports Medicine (2012) on behalf of other organizations and experts – organized PA benefits in six dimensions: physical capital, including physical health (e.g., motor skills, cardiorespiratory fitness, muscular strength, and bone/joint health) and prevention of diseases; emotional capital, encompassing psychological benefits related to satisfaction, self-esteem, and self-efficacy as well as prevention and treatment of depression and anxiety; individual capital, focusing on character-related elements (e.g., life skills, sportsmanship, time management, or commitment); social capital, including strengthening of social networks through trust, collaboration, or teamwork, as well as reduction of crime; intellectual capital, encompassing cognitive-related gains (e.g., executive functions, attention, academic achievement) and management of learning disabilities and cognitive decline; and financial capital, including job-related gains (e.g., productivity and income), as well as reduced costs of health care and absenteeism/presenteeism. It should, however, be noted that not all forms of PA deliver the same benefits. Given their multiple demands, sports, in particular open skill sports – characterized by constantly changing conditions, to which movements must be flexibly adapted – may result in maximized benefits. This is the case of team sports and martial arts.

The most popular team sport is football (or soccer). Several studies support the widespread benefits of football throughout the lifespan (Krustrup et al., 2010). A systematic review conducted by Oja et al. (2015) revealed that football was one of the most beneficial sports for adults in terms of cardiovascular and metabolic health (see also Zouhal et al., 2020). Psychological-related benefits have also been reported, mainly in cognitive dimensions. Verburgh et al. (2014) found that highly talented soccer players (aged 8–12) surpassed amateurs in motor inhibition and attentional skills. Chen et al. (2019) found that football improved visuo-spatial working memory in young adults without intellectual disabilities as well as sports motivation and attention in their disabled peers. These findings indicate that football requires more than athletic and tactical skills (Vestberg et al., 2012; Verburgh et al., 2014): as a complex and quickly changing context, football requires players to be able to rapidly adapt, change strategy, and inhibit responses; as a team sport, it requires players to socially interact, cooperate with teammates, and anticipate other players’ behaviors and ball movements.

Karate, a recently Olympics-approved sport, is a very dynamic, holistic, and popular Japanese hard martial art (Nakayama, 1976), combining mental and spiritual development with physical strength, speed, and endurance to produce powerful, fast, and vigorous striking movements (Theeboom and Knop, 1999). The practice of hard martial arts, including karate, has been associated with physical health benefits, such as improvements in postural control, muscular strength and/or skeletal status, and cardiovascular fitness (Rios et al., 2018). Karate athletes were also found to be among the martial artists with the greatest intensity of health behaviors (Kotarska et al., 2019). The psychological benefits of karate are also becoming known. Elite karateka displayed better perceptual, visual, and attention skills than amateurs and non-practitioners (Russo and Ottoboni, 2019). Children with 3–5 years of karate experience displayed better executive functions than their peers (Alesi et al., 2014). The practice of karate also reduced anxiety and increased processing speed and mental health in elders (Jansen et al., 2017), and improved socioemotional skills in children with autism spectrum disorders (Movahedi et al., 2013). The widespread benefits of karate might seem related to its multidimensional nature. Likely, karate cognitive benefits rely on its high motor and cognitive demands (Diamond, 2015), whereas socioemotional ones arise from karate focus on body awareness, dyadic interactions, and moral values (Vertonghen and Theeboom, 2010; Rassovsky et al., 2019).

Despite the benefits, the practice of sports is not devoid of risks (American College of Sports Medicine, 2014). A particularly controversial risk is the exhibition of aggressive behaviors (Wann, 2005). Aggressive actions among athletes can be seen as an integral part of many sports, needed for winning (Fitch and Marshall, 2001). This is evident in team contact sports – such as ice hockey, rugby, or football. Traclet et al. (2015) found a shared aggression norm in football, though to a lesser extent than in ice hockey. Though commonly labeled as “combat sports,” Eastern traditional martial arts, aimed at developing fighting skills as much as non-violent attitudes, lack such norm (Theeboom and Knop, 1999; Klimczak et al., 2014). Indeed, meta-analytic findings support an association between the practice of martial arts and a reduction in aggressive tendencies (Harwood et al., 2017). Moreover, Sofia and Cruz (2013) found that football players reported higher levels of aggressiveness and anger than kickboxing and self-defense athletes.

All in all, the benefits and aggression-related risks of sports in general, and of football and karate in particular, are coming to light. However, though empirical evidence showing these benefits or risks are certainly important, people’s intentions to participate in sports mostly rely on their own beliefs.

According to the Theory of Planned Behavior (TPB), key antecedents of people’s intentions to engage in a behavior are attitudes, subjective norms, and perceived behavioral control (Ajzen, 2012; Bosnjak et al., 2020). A meta-analysis found that, along with perceived behavioral control (i.e., people’s beliefs about factors influencing their behavioral engagement), another powerful influence in forming intentions to participate in PA is people’s attitudes, that is, their beliefs about the positive and negative consequences of practicing that activity (Hagger et al., 2002). Favorable attitudes are associated with stronger intentions to perform a behavior and, when the opportunity arises, to carry it out (Ajzen, 2012). Thus, in addition to other factors influencing intention to participate in PA, such us perceived behavioral control, people’s perceptions of benefits and risks will influence their intention to participate in PA.

Past research already recognized the importance of measuring people’s attitudes toward PA, but this can be characterized by a general measurement approach to both attitudes and PA (Macgregor et al., 2017). Grounded on the TPB (Hagger and Chatzisarantis, 2005), attitudes have been assessed through a set of 6-point semantic differential items about participation in PA from affective (e.g., enjoyable-unenjoyable) and instrumental (e.g., important-unimportant) stands. However, moving away from the typical TPB methodological approach, some researchers have narrowed the concept of attitudes to perceived benefits of PA participation and related this construct with effective (rather than intended) participation in PA. These studies used either unidimensional scales combining physical and multiple psychological benefits (Booth et al., 2000; Dergance et al., 2003; Cardenas et al., 2009; King et al., 2014; Roth et al., 2019), or bidimensional scales splitting between the two (Patel et al., 2013). A handful of studies have focused on the perceived benefits of specific sports, mainly among athletes. Mason and Holt (2018) showed that adults with severe mental illness perceived both physical and psychological gains in football. Moreover, Barfield and Malone (2013) characterized the perceived benefits to exercise among power wheelchair soccer players, using a bidimensional scale tapping personal and environmental benefits. In the field of martial arts, studies on market demands used unidimensional scales targeting either psychological (Kim et al., 2009), or physical and psychological benefits (Kim and Zhang, 2019). Rogowska and Kuśnierz (2013) also used a measure combining cognitive, behavioral, and affective dimensions.

Despite their contribution to the field, the above-cited studies were limited in two ways. First, all studies failed to theoretically and/or statistically discriminate between different psychological benefits (e.g., social vs. emotional). The majority of these works performed preliminary factorial analyses that grouped together items tapping different types benefits (e.g., Booth et al., 2000; Cardenas et al., 2009; Kim et al., 2009; Patel et al., 2013). On the one hand, this can be explained by the inclusion of other dimensions besides benefits in the analysis. For example, Kim et al. (2009) conducted a confirmatory factor analysis with 17 latent factors, with perceived benefits being only one of them. On the other hand, it can be related to the reduced and/or unbalanced number of items tapping the different types of benefits. For instance, Cardenas et al. (2009) used a scale with 13 items mainly tapping physical benefits, with only four items targeting socio-emotional aspects. Second, the majority of these works left out well-known psychological benefits (typically, cognitive ones), raising questions about instruments’ content validity. Even the study of Rogowska and Kuśnierz (2013), which included an instrument with a component labeled “cognitive,” limited its focus to the measurement of respondents’ perceptions of knowledge about martial arts. An exception to these studies, was the work of Lakes et al. (2016), who developed a scale to measure dancers’ perceptions of the physical, cognitive, emotional, and social benefits of partnered dancing. Confirming the importance of discriminating among different benefits, authors reported that perceived benefits in these dimensions varied as a function of dancers’ experience and commitment as well as length and frequency of participation.

Some of the previously cited studies also focused on barriers to participation related to PA negative outcomes (or risks), such as fear of injury (Booth et al., 2000; Dergance et al., 2003; Patel et al., 2013). Still, none of them identified aggression-related issues as barriers to either PA or sports participation. Only Rogowska and Kuśnierz (2013) found that negative or weak attitudes toward martial arts were present among people who saw brutality as the dominant feature of martial arts. A relevant study targeting perceived aggression in sports was that of Pedersen (2007), who asked 285 non-athletes college students to provide perceived aggressiveness ratings on 16 sports (excluding martial arts). In ascending order, the top 5 were: wrestling, football, rugby, boxing, and hockey. Additionally, higher and lower perceived aggression was associated with lower and higher ratings in willingness to participate, respectively. These non-athletes’ perceptions were aligned with those provided by competitive athletes (Maxwell, 2007). Overall, the study of perceived aggression associated with sports as received little research attention. However, to study these perceptions is particularly relevant, as they may represent a significant barrier to engagement in sports, in some cases, ungrounded. For example, despite the non-violent attitudes that lie at the heart of karate practice, if people perceive karateka as more prone to exhibit aggressive behaviors, not only inside but also outside the dojo, their intention to practice it may be reduced.

## Present Study

This article focused on undergraduates perceived benefits and aggression-related risks of two widely practiced sports, known to bring widespread benefits to athletes: karate and football. Although the actual benefits (and some risks) of these sports are known, past works have neither compared people’s perceptions about karate and football, nor examined how past/present participation in different forms of PA (including but not limited to sports) may shape them. Perceived benefits and risks are likely to vary across sports with different features, such as karate and football, which may, in turn, differently influence individuals’ interests, values, and behaviors toward those sports. Information on karate and football perceptions can be used to boost involvement in these sport activities. This is even more relevant in younger adults, who will shortly make choices for their children.

This study was conducted with a twofold goal. First, we aimed to develop and examine the validity and reliability of a scale to measure perceived physical, emotional, cognitive, and social benefits as well as perceived aggression-related risks of karate and football. For that, we examined the factorial validity of the scales, inspected factors’ reliability, and tested their convergent/discriminant validity. Second, we intended to compare perceived benefits and risks within and between karate and football, as well as among undergraduates with past/present involvement in different types of PA. Grounded on the previously surveyed literature, we expected to find differential perceptions between these sports and among undergraduates with different types of involvement in PA.

## Materials and Methods

### Participants’ Characterization

This study included a convenience sample of 184 undergraduate students in Psychology (86% females) with a mean age of 20.25 years (*SD* = 3.49, range = 17–47). Among these, 139 undergraduates reported a present or past participation in PA (hereafter referred as PA participants), whereas 45 did not (24%). The categorization and distribution of mentioned PA, including average years of practice and training frequency is presented in Table 1. On average, each PA participant referred to have practiced two different activities for an average duration of 4.59 years. The training frequency of these PA was distributed as follows: 54% had low training frequency (1–2 days/week), 36% had moderate frequency (3–4 days/week), and 10% had high training frequency (5–7 days/week). Swimming was the most frequent PA, with 52% of the sample referring to have practiced this PA for an average of 5 years, followed by team sports (42%, average of 4 years of practice), dance (34%, average of 6 years of practice), and martial arts (27%, average of 3 years of practice). Among the undergraduates who have said to be involved in team sports (*n* = 59) or martial arts (*n* = 37), the most frequently mentioned type of sport was football (*n* = 23; 59%) and karate (*n* = 16, 43%), respectively. Football was practiced for an average of 4 years, with 35% of football players reporting a low training frequency and 61% a moderate training frequency. Karate was practiced for an average of 3.5 years, with 75% of karateka reporting a low training frequency, 19% a moderate training frequency, and 6% a high training frequency.

**TABLE 1:** Characterization of the sample in terms of physical activity (PA) practiced (*n* = 139).

	Number and Percentage of Practitioners		Years of Practice	Training Frequency			
Type of PA				Low	Moderate	High	No response
Swimming	72	51.80%	4.85	49	17	5	1
Team sports	59	42.45%	3.91	21	30	6	2
Dance	47	33.81%	5.95	26	16	5	0
Martial arts	37	26.62%	3.37	24	12	1	0
Racquet sports	17	12.23%	4.13	10	6	0	1
Gymnastics	16	11.51%	4.04	8	4	3	1
Gym workout routines	13	9.35%	2.68	2	9	2	0
Skating sports	11	7.91%	3.50	3	4	4	0
Horseback riding	8	5.76%	5.14	7	0	1	0
Yoga/Pilates	7	5.04%	4.08	4	3	0	0
Athletics	6	4.32%	3.83	3	2	1	0
Cycling	2	1.44%	23.00	0	1	1	0
Golf	1	0.72%	7.00	1	0	0	0

### Perceived Benefits and Aggressiveness Risks Scale (PBAR Scale)

As recommended by Boateng et al. (2018), the development of the scale involved (1) *a priori* identification of the domains to be measured, which was based on a thorough literature review; and (2) generation of the items to measure each domain, which followed a deductive method and was grounded on the literature review used to identify the domains as well as on the inspection of comparable scales. Further details are provided below, separately for the benefits and aggressiveness-related risk factors.

Based on TPB, we defined perceived benefits as the positive consequences arising from practice (Ajzen, 2012). According to literature on PA benefits (for a review see American College of Sports Medicine, 2012), perceived benefits were organized into four domains: physical, cognitive, emotional, and social. Four items were generated for each domain, in line with empirically based effects of sports in general and karate and football in particular, and inspired by other instruments, namely, the bi-dimensional scale of Kim and Zhang (2019) measuring psychological and physical benefits in martial arts; the uni-dimensional scale of Kim et al. (2009) tapping personal benefits in taekwondo; the multidimensional scale of Lakes et al. (2016) assessing physical, cognitive, emotional, and social benefits in partnered dancing; and the benefits sub-scale of the parent perceptions of PA developed by Lakes et al. (2019). Physical benefits focused on disease prevention as well as improvement of body posture, muscular strength, and motor coordination (Oja et al., 2015; Rios et al., 2018; Kotarska et al., 2019; Zouhal et al., 2020). Cognitive benefits included increases on learning skills, attention, and school/work achievement (Vestberg et al., 2012; Chen et al., 2019; Russo and Ottoboni, 2019). Emotional benefits targeted anxiety, emotional regulation, self-esteem, and well-being (Movahedi et al., 2013; Jansen et al., 2017; Chen et al., 2019). Social benefits tapped cooperation skills, respect for others, sense of belonging, and moral values (Movahedi et al., 2013; Rassovsky et al., 2019).

Given our interest in studying people’s perception of aggression-related risks resulting from the practice of karate and football, a fifth domain targeting this (putative) negative outcome was created. This domain was defined as the use of physical or verbal violence to achieve goals (Arriaga et al., 2004). Based on past work on aggression in general and on aggression in sport (Fitch and Marshall, 2001; Wann, 2005; Traclet et al., 2015), and the physical and verbal aggression sub-scales of the Aggression Questionnaire (Buss and Perry, 1992), we generated four items focused on physical and verbal aggressiveness, use of violence, and exaggerated competitive attitudes.

This procedure resulted in a 5-factor scale composed of 20 items, which were exactly the same for the karate and football versions. Although this study targeted undergraduates, language was formulated having in mind the possibility of testing and using this scale with youngsters in the future. The first Portuguese version of the PBAR scale was elaborated by the first author. This was then shared with a group of experts composed by a 7th-dan karate sensei (second author), a football coach, and an educational psychologist. The group was presented with the overall goal of the scale and specific definitions of the targeted domains along with the items. Then, they were asked to rate from 1 (*not very well*) to 5 (*very well*) the degree to which the instrument was aligned with its goal, and the degree to which items were representative of the potential benefits or aggressive-related outcomes arising from sport practice in general, and karate and football in particular. Experts were also asked to accept, reject, or modify items in terms of their appropriateness to measure the respective domain and in terms of linguistic formulation. Confirming the scale’s face and content validity, experts agreed that the scale was “very well” aligned with its purpose and that the items represented “very well” the targeted outcomes in each domain. Moreover, all items were accepted with minor suggestions concerning language. This input was used to fine-tune the PBAR scale and achieve its final version.

Examples of items taping karate/football perceived outcomes are: “Turns muscles stronger” (physical benefit); “Improves capacity for concentration and work” (cognitive benefit); “Promotes feelings of well-being and satisfaction with life” (emotional benefit); “Stimulates respect for other persons” (social benefit); and “Favors the use of violence to solve problems” (aggressiveness-related risk).

### Procedure

The sample was recruited during mandatory undergraduate Psychology classes. After a brief presentation of the study, undergraduates were told that participation in the study would take no more than 10 min, would be fully anonymous and voluntary, and no incentives would be offered. Undergraduates who accepted to collaborate were asked to fill in the PBAR in relation to karate and football in group. For that, they should indicate the degree to which they perceived a set of statements to represent consequences of the regular practice of karate and football, using a 5-point scale from 1 (*totally disagree*) to 5 (*totally agree*). Then, they were asked to indicate gender and age, and whether they practiced or were currently practicing any type of PA. If yes, participants should name the activity and indicate years of practice and training frequency. The study was approved by the ethical committee of the first author institution.

### Data Analysis Strategy

Before conducting the analyses, we checked evidence of common method bias using the Harman’s single-factor test (Podsakoff et al., 2003). After loading all items into a common factor, we examined if the amount of explained variance was above 50%, which would be evidence of method bias. Results showed that the single component accounted for 16% of the covariance between all items, indicating that common method bias was not a concern in the present study.

#### Goal 1: Test of PBAR’s Karate and Football Versions

Two confirmatory factorial analyses (CFA) were conducted to examine the factorial structure of the karate and football versions of the PBAR scale, using the R system for statistical computing (R Development Core Team, 2018). Latent variables were scaled by imposing unit of loading identification constraints. The variance of all latent factors was constrained to equal 1.0, so that all factor loadings could be freely estimated. Based on the recommendation from Kline (2016), we used the following indexes to evaluate model fit: chi-square statistic (χ^2^) along with χ^2^/*df* statistic, confirmatory fit index (CFI), root-mean-square error of approximation (RMSEA), and standardized root mean residual (SRMR). χ^2^/df values < 2 and 3, CFI values > 0.95 and 0.90, RMSEA values < 0.06 and 0.10, and SRMR values < 0.06 and 0.09 are considered good and adequate fits, respectively (Hu and Bentler, 1999; Schermelleh-Engel et al., 2003). Additionally, we examined factor loadings, reliability coefficients (via the ordinal omega coefficient; see Revelle and Zinbarg, 2009; Dunn et al., 2014), and inter-item correlations for each factor. This information was analyzed for the karate and football versions and used to drop items not working as expected in both versions, so a single scale with the same items could be achieved. The same CFA and reliability analyses were then conducted to examine the adequacy of the reduced version.

Finally, we examined two forms of convergent and discriminant validity, namely, within and between the karate and football versions of the PBAR. First, for both versions separately, we made a stringent test of their internal structure by computing the average variance extracted (AVE), with values above 0.50 indicating good convergent validity for each factor; and we compared the AVE of each factor with the squared correlation of that with other factors, in which an higher AVE indicates good discriminant validity between factors (Hair et al., 2010). Second, we examined the degree to which there were higher associations between the same factors of the two versions than between different factors (Furr, 2011). For that, we correlated all factors of the karate and football versions (Pearson’s correlations) and computed average correlations using the Fisher *Z* transformation.

#### Goal 2: Test of PBAR’s Karate and Football Versions

To examine differences between karate and football perceptions and among different types of PA participants, we conducted a 2 (Sport [karate, football]) × 5 (Perceptions [physical benefits, emotional benefits, social benefits, cognitive benefits, aggressiveness risks]) × 4 (PA participants [martial artists, team sports players, participants in other types of PA, and non-participants]) Analysis of Variance, with repeated measures in the first two factors. Significant interactions were examined with tests of simple effects. When significant, these were followed-up through pairwise comparisons with Bonferroni correction.

## Results

### Goal 1: Test of PBAR’s Karate and Football Versions

The CFA on the 20-item PBAR scale revealed an inadequate model fit for the karate version, χ^2^(160, *N* = 184) = 321.023, χ^2^/*df* = 2.01, CFI = 0.767, RMSEA = 0.074, SRMR = 0.080, but an adequate (though with room for improvement) model fit for the football version, χ^2^(160, *N* = 184) = 241.442, χ^2^/*df* = 1.51, CFI = 0.896, RMSEA = 0.053, SRMR = 0.065. Reliability estimates ranged from 0.54 to 0.76 and 0.60 and 0.78, respectively, in the karate and football versions. We then looked into each factor to identify the items with the lowest factor loadings and lowest inter-item correlations in both versions. Based on this scrutiny, we identified one item per factor that was working poorly in both versions. These items were removed and the 15-item PBAR scale was then examined.

CFA results concerning the shortened scale revealed adequate-to-good model fits for the karate, χ^2^(80, *N* = 184) = 119.937, χ^2^/*df* = 1.49, CFI = 0.920, RMSEA = 0.052, SRMR = 0.063, and football versions, χ^2^(80, *N* = 184) = 126.936, χ^2^/*df* = 1.59, CFI = 0.924, RMSEA = 0.056, SRMR = 0.061. Table 2 presents descriptive statistics for all items and factors, including factor loadings. These ranged from 0.26 to 0.88 and 0.40 to 0.81, respectively, in the karate and football versions (all *p*s < 0.003). Respectively, reliability estimates (ω) for the karate and football versions were: 0.69 and 0.71 for physical benefits, 0.64 and 0.68 for emotional benefits, 0.60 and 0.54 for social benefits, 0.60 and 0.63 for cognitive benefits, and 0.82 and 0.80 for risks. Despite the acceptable factor loadings, AVE was below 0.50 for all factors (range = 0.27–0.32 for the karate version and 0.25–0.40 for the football version), except for the aggression-risk factors, where AVE was 0.58 for both versions. Confirming good discriminant validity, the squared correlations between factors were below AVE values for each factor. Complete results are presented in Table 3.

**TABLE 2:** Descriptive statistics, including factor loadings, of the retained karate and football items.

		Karate							Football						

		*Min.*	*Max.*	*M*	*SD*	*Sk*	*Ku*	λ	*Min.*	*Max.*	*M*	*SD*	*Sk*	*Ku*	λ
Physical Benefits		3.00	5.00	4.52	0.47	–0.78	–0.14		3.33	5.00	4.61	0.43	–0.84	–0.28	
	Item 6	1.00	5.00	4.35	0.80	–1.31	2.22	0.56	1.00	5.00	4.58	0.70	–2.26	7.20	0.42
	Item 14	2.00	5.00	4.46	0.64	–1.04	1.11	0.50	3.00	5.00	4.62	0.53	–0.97	–0.19	0.49
	Item 20	3.00	5.00	4.74	0.50	–1.79	2.40	0.49	2.00	5.00	4.64	0.60	–1.59	2.17	0.57
Cognitive Benefits		2.67	5.00	4.00	0.52	–0.15	–0.08		2.00	5.00	3.55	0.62	–0.09	–0.28	
	Item 1	2.00	5.00	4.37	0.59	–0.63	1.31	0.44	1.00	5.00	3.65	0.83	–0.38	–0.04	0.55
	Item 9	1.00	5.00	3.68	0.80	–0.16	–0.05	0.59	1.00	5.00	3.41	0.80	0.02	0.17	0.61
	Item 16	2.00	5.00	3.93	0.74	–0.38	–0.02	0.59	1.00	5.00	3.59	0.82	–0.25	–0.11	0.67
Emotional Benefits		2.67	5.00	4.25	0.50	–0.41	–0.19		2.00	5.00	3.73	0.62	–0.30	–0.03	
	Item 7	2.00	5.00	4.21	0.78	–0.80	0.34	0.52	1.00	5.00	3.04	0.92	0.22	–0.45	0.51
	Item 15	2.00	5.00	4.26	0.67	–0.46	–0.31	0.59	1.00	5.00	3.98	0.83	–0.60	0.25	0.64
	Item 19	3.00	5.00	4.28	0.61	–0.24	–0.60	0.53	2.00	5.00	4.18	0.69	–0.46	–0.09	0.73
Social Benefits		2.00	5.00	3.93	0.64	–0.56	0.21		1.67	5.00	3.65	0.65	–0.29	–0.05	
	Item 2	2.00	5.00	4.20	0.77	–0.72	0.08	0.72	1.00	5.00	3.23	1.00	–0.05	–0.85	0.58
	Item 10	1.00	5.00	3.54	1.04	–0.29	–0.65	0.26	1.00	5.00	4.51	0.67	–1.57	3.91	0.40
	Item 13	1.00	5.00	4.06	0.94	–0.99	0.76	0.61	1.00	5.00	3.21	0.99	0.05	–0.59	0.67
Aggressiveness Risks		1.00	4.67	2.16	0.82	0.39	–0.18		1.00	5.00	3.23	0.92	0.06	–0.69	
	Item 8	1.00	5.00	2.22	1.04	0.56	–0.34	0.79	1.00	5.00	2.60	1.10	0.26	–0.69	0.77
	Item 11	1.00	5.00	2.27	1.05	0.72	0.04	0.88	1.00	5.00	2.40	1.00	0.34	–0.56	0.81
	Item 18	1.00	4.00	1.98	0.85	0.47	–0.51	0.58	1.00	5.00	3.30	1.15	–0.38	–0.82	0.71

**TABLE 3:** Bivariate correlations between karate and football perceived benefits and risks (same-factor correlations in bold).

PBAR Versions and Factors		AVE	MSV	Karate					Football			
				1	2	3	4	5	6	7	8	9
**Karate**												
	(1) Physical Benefits	0.27	0.04									
	(2) Cognitive Benefits	0.30	0.21	0.15*								
	(3) Emotional Benefits	0.30	0.21	0.21**	0.46***							
	(4) Social Benefits	0.32	0.19	0.21**	0.31***	0.44***						
	(5) Aggressiveness Risks	0.58	0.13	–0.09	–0.16*	–0.30***	–0.36***					
**Football**												
	(6) Physical Benefits	0.25	0.12	0.69***	0.23***	0.17*	0.16*	–0.01				
	(7) Cognitive Benefits	0.37	0.28	0.24***	0.52***	0.18*	0.13	–0.02	0.31***			
	(8) Emotional Benefits	0.40	0.26	0.31***	0.18*	0.49***	0.18*	–0.08	0.33***	0.40***		
	(9) Social Benefits	0.32	0.26	0.23**	0.28***	0.14	0.22**	–0.02	0.34***	0.53***	0.51***	
	(10) Aggressiveness Risks	0.58	0.16	0.20**	0.02	0.03	0.04	0.47**	0.17*	0.25***	0.40***	0.33***

AVE, average variance extracted. MSV, maximum shared variance (computed separately for each version). *p < 0.05, **p < 0.01, ***p < 0.001.

Concerning the associations between factors of the two versions, also displayed in Table 3, results showed that correlations between karate factors ranged from 0.09 to 0.46, with an average of 0.27; and correlations between football factors ranged from 0.17 to 0.53, with an average of 0.36. Correlations between the same karate and football factors ranged from 0.22 to 0.69, with an average of 0.49; whereas those between different karate and football factors ranged from 0.01 to 0.31, with an average of 0.14.

### Goal 2: Comparison of Karate and Football Perceptions

Table 4 shows means and standard deviations for karate and football perceived benefits and risks by type of PA participant.

**TABLE 4:** Perceived benefits and aggressiveness risks of karate and football by type of participation in PA.

		Martial Artists		Team Sports Players		Participants in Other PA		Non-participants in PA	
		*n* = 30		*n* = 44		*n* = 65		*n* = 45	
Karate		*M*	*SD*	*M*	*SD*	*M*	*SD*	*M*	*SD*
	Physical Benefits	4.44	0.48	4.59	0.48	4.55	0.48	4.45	0.43
	Cognitive Benefits	4.02	0.55	4.04	0.52	4.03	0.41	3.90	0.62
	Emotional Benefits	4.34	0.47	4.26	0.49	4.31	0.49	4.07	0.52
	Social Benefits	4.17	0.51	3.95	0.61	3.86	0.65	3.88	0.71
	Aggressiveness Risks	1.83	0.74	2.01	0.92	2.23	0.81	2.42	0.70
**Football**									
	Physical Benefits	4.48	0.48	4.67	0.44	4.64	0.40	4.61	0.42
	Cognitive Benefits	3.39	0.64	3.64	0.72	3.58	0.56	3.52	0.59
	Emotional Benefits	3.57	0.62	3.80	0.69	3.78	0.66	3.71	0.45
	Social Benefits	3.33	0.52	3.72	0.78	3.74	0.70	3.65	0.45
	Aggressiveness Risks	3.20	0.90	3.38	0.97	3.22	0.95	3.13	0.86

**FIGURE 1:**
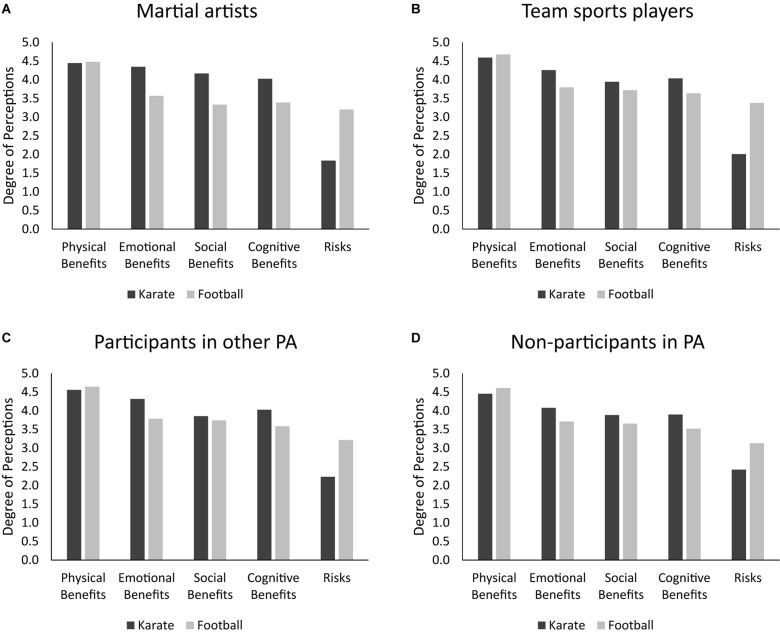
Illustration of the perceptions × sport × type of PA participation interaction. Each panel depicts karate and football perceptions for different types of PA participants: **(A)** martial artists, **(B)** team sport players, **(C)** participants in other PA, and **(D)** non-participants in PA.

Results revealed a main effect of Perceptions, *F*(4,720) = 486.64, *p* < 0.001, η_*p*_^2^ = 0.73, an interaction between Sport and Perceptions, *F*(4,720) = 124.81, *p* < 0.001, η_*p*_^2^ = 0.41, and a three-way interaction, *F*(12,720) = 3.14, *p* < 0.001, η_*p*_^2^ = 0.05. This latter is depicted in Figure 1 and further detailed below.

#### Differences Between Sports

Participants in other PA and non-participants perceived more physical benefits in football than in karate, *F*s(1,180) > 3.90, *p*s < 0.05, η_*p*_^2^ > 0.02; however, martial artists and team sports players perceived similar physical benefits in karate and football, *F*s(1,180) < 2.42, *p*s > 0.12, η_*p*_^2^ < 0.02. Moreover, martial artists, team sports players, and participants other PA, *F*s(1,180) > 3.79, *p*s < 0.05, η_*p*_^2^ > 0.02, but not non-participants, *F*(1,180) = 1.51, *p* = 0.22, η_*p*_^2^ = 0.01, perceived karate to have more social benefits than football. For the whole sample, karate was perceived to have more cognitive and emotional benefits as well as less aggression-related risks than football, *F*s(1,180) > 10.12, *p*s < 0.001, η_*p*_^2^ > 0.05.

#### Differences Between Perceptions

All types of PA participants saw differences between benefits/risks in karate as well as in football, *F*s(4,177) > 32.29, *p*s < 0.001, η_*p*_^2^ > 0.42. Concerning karate: (a) martial artists perceived more physical than cognitive benefits (*p* = 0.004), but similar physical, emotional, and social benefits (*p*s > 0.30), whereas all others perceived physical benefits to surpass all other benefits (*p*s < 0.02); (b) only martial artists and participants in other PA perceived more emotional than cognitive benefits (*p*s < 0.01), and only team sports players and participants in other PA perceived more emotional than social benefits (*p*s < 0.01); (c) the whole sample perceived aggression-related risks to be lower in comparison to benefits (*p*s < 0.001). Regarding football: (a) the whole sample perceived physical benefits to be greater than all other benefits and risks (*p*s < 0.001) and emotional benefits to be of the same extent as social and cognitive benefits (*p*s > 0.19); (b) except participants in other PA (*p* = 0.03), all others perceived similar cognitive benefits and aggressiveness risks (*p*s > 0.08); (c) participants in other PA and non-participants perceived emotional and social benefits to be higher than risks (*p*s < 0.002), whereas team sports players perceived more emotional but not social benefits than risks (*p*s = 0.02 and 0.16, respectively); (d) martial artists perceived aggression-related risks to be of the same extent as emotional and social benefits (*p*s > 0.24). Regardless of PA participants type, cognitive benefits were perceived to the same extent as social benefits, both in karate and football (*p*s > 0.46).

#### Differences Between Type of PA Participants

In general, perceived benefits and aggressiveness risks of karate and football were similar across all types of PA participants, *F*s(3,180) < 2.62, *p*s > 0.05, η_*p*_^2^ < 0.04. There were however two exceptions concerning karate risks, *F*(3,180) = 3.96, *p* = 0.01, η_*p*_^2^ = 0.06, and football social benefits, *F*(3,180) = 3.02, *p* = 0.03, η_*p*_^2^ = 0.05. Specifically, martial artists perceived less aggression-related risks in karate than non-participants (*p* = 0.01) and less social benefits in karate than participants in other PA (*p* = 0.03).

## Discussion

This study had two major goals: to develop and test a scale to measure perceived benefits and aggressiveness risks (PBAR scale), and to compare those perceptions between karate and football, and among participants in different types of PA.

### Goal 1: Test of PBAR Karate and Football Versions

Based on past works, we developed the PBAR scale to measure perceived physical, emotional, cognitive, and social benefits along with perceived aggression-related risks in karate and football. After dropping five items, we confirmed the factorial validity of the 15-item instrument. In both versions, we found acceptable factor loadings and reliability indices. Still, the Social Benefits factor worked poorer than the others, which should be kept in mind when interpreting current findings. More tests on the instrument seem therefore needed, with particular attention to that factor, as it may require additional fine-tuning. It should additionally be noted that, except the aggression-related risk factors, all others failed to achieve satisfactory convergent validity (AVE < 0.50). Even though our findings supported the factorial validity of both scales, these less than perfect AVE estimates should not be overlooked. They can be related to the heterogeneity of our sample, which included undergraduates with varying degrees of PA participation, who may have interpreted items differently, thus resulting in more error than explained variance. For example, though the majority of the sample reported a past or present involvement in PA, 24% of the surveyed undergraduates have no prior participation in any kind of PA. Future studies should test these scales with more homogeneous samples and inspect whether AVE-related issues disappear or if items modifications are warranted.

Findings were encouraging concerning the degree to which the different factors discriminated among different perceptions. In line with the premise that gave raise to this study, perceived physical, emotional, cognitive, and social benefits were distinguishable outcomes of karate and football. Thus, for a fine-grained analyses of sports perceived benefits, it seems advisable to use multidimensional rather unidimensional scales, as done before (Kim et al., 2009; Barfield and Malone, 2013; Kim and Zhang, 2019). Further supporting this conclusion, results on the convergent/discriminant validity between the two versions of the PBAR karate and football versions were also as expected. Within each version, factors were generally correlated with each other. Across versions, there were higher correlations between the same factors, and lower correlations between different factors.

All in all, findings provided preliminary evidence on PBAR validity and reliability. However, further tests seem needed to gather more evidence on its psychometric properties. For instance, it would be important to examine the instrument stability over time (test–retest reliability), to study the degree to which it predicts intention to participate in the targeted sport (predictive validity), or to test the scale’s ability to detect change, for example, after raising people’s awareness of sports’ real benefits/risks (responsiveness to change). These future tests should consider including larger samples, preferably estimated using *a priori* power analysis. This was not the case of the present study, even though observed power was above 0.80. Additionally, it would be important to test the karate and/or football versions of the PBAR with different populations and test for measurement invariance, for example, contrasting different age groups (e.g., adolescents vs. adults), types of athletes (e.g., karateka vs. footballers), or expertise levels (i.e., beginners vs. advanced). Finally, it could also be valuable to test the PBAR in the context of other sports, besides football and karate.

### Goal 2: Comparison of Karate and Football Perceptions

As anticipated, perceived benefits and aggression-related risks varied between and within the sports targeted, as well as across type of PA participant. Results showed that martial artists perceived karate to bring similar physical, emotional, and social benefits, whereas all others perceived physical benefits as the main outcome of karate. For the whole sample, physical benefits were recognized as the most salient benefit of football, with emotional, social, and cognitive benefits being perceived to the same extent. The general strongest perception of physical benefits in karate and football aligns with several studies reporting real health benefits of these modalities (Oja et al., 2015; Rios et al., 2018; Kotarska et al., 2019; Zouhal et al., 2020). The perceptions of martial artists were particularly interesting, as they recognized that karate brings as much physical as emotional and social benefits. The real socioemotional outcomes of martial arts, including karate, have already been reported (Movahedi et al., 2013; Jansen et al., 2017). Due to their own experience, martial artists may be more cognizant of these benefits than people who never tried any martial art and, likely, have a reduced knowledge about it. The result that cognitive benefits were not seen as a salient outcome of karate and football is surprising, given the increasing amount of research documenting the cognitive benefits of these sports (Alesi et al., 2014; Verburgh et al., 2014; Chen et al., 2019; Russo and Ottoboni, 2019). There seems to be a mismatch between real and perceived cognitive benefits in karate and football, which calls for more research attention.

This is the first study providing comparative data on football and karate perceptions. A main finding was that martial artists and team sports players saw similar physical benefits in these activities, whereas participants in other PA and non-participants perceived more physical benefits in football than karate. Martial artists and team sports players own experience with these or related sports, along with an eventual lack of knowledge among the others, may explain this difference (Lakes et al., 2016). In general, our sample perceived karate to bring more psychological-related benefits than football. This finding is not surprising as it may reflect the nature of karate. In addition to increasing physical skills (e.g., strength, speed, coordination), karate practice is aimed at developing karateka’s mind and spirit (Theeboom and Knop, 1999; Vertonghen and Theeboom, 2010; Rassovsky et al., 2019). More than being a sport, as a traditional martial art, karate is a way of life. Karateka develop their ability to engage in states of awareness and openness to surrounding threats (*zanshi*) and states of flow totally focused on the activity (*mushin*), while following five moral principles (*dojo kun*): seek perfection of character, be faithful, endeavor to excel, respect others, and refrain from violent behavior (Nakayama, 1976). Though football may also positively affect some of these aspects, this may be more a by-product than the main goal of practice.

It is worth noticing that our study showed that perceived benefits of karate and football varied as a function of undergraduates’ characteristics, specifically, their past/present engagement in different types of PA. This result is not new, as past studies already reported PA benefits to vary across participants gender, ethnicity, body size (Roth et al., 2019), degree of PA activity (Cardenas et al., 2009), or experience, commitment, and degree of participation in PA (Lakes et al., 2016). Further research is, however, needed to examine whether karate and football perceived benefits differ among athletes with varying expertise levels (e.g., beginning, intermediate, and advanced) and the factors underlying those differences (e.g., greater knowledge, personal experience, or biased perceptions toward a valued modality).

With respect to aggression-related risks, these were perceived to be lower in karate than in football. This finding is in line with past works that found higher levels of reported aggressiveness and anger among football players than athletes engaged in martial arts related activities, such as kickboxing and self-defense (Sofia and Cruz, 2013). Moreover, this finding extends the results of Pedersen (2007). Despite not including martial arts in their analysis, they found football to be among the sports with the highest perceived levels of aggressiveness among undergraduates. Our results also showed that the perceived benefits of karate clearly outweighed its aggressiveness risks. Karate does not seem to carry negative connotations, such as deeming this martial art as dangerous or instigator of aggressive behaviors. In line with TPB (Ajzen, 2012; Bosnjak et al., 2020), the lack of such connotation along with the benefits ascribed to karate is certainly a first step to have people engaged in this sport. This positive attitude among younger adults is particularly encouraging as it may not only be an incentive for themselves to get involved in karate, but also a key driver to engaging their children. Given the documented benefits of this martial art, having youngsters practicing karate can be means to complement their education and build physically and mentally strong members of the society. Contrary to karate, the perceived psychological-related benefits of football did not clearly outweigh the perceived aggression-related risks of this modality. This is a curious finding because, despite its noticeable risks, which go beyond aggressiveness (see for example Schatz et al., 2020 on perceived concussion risks), football is the number one sport in the world, with 265 million active footballers in 2006 (Kunz, 2007).

Three caveats should, however, be kept in mind concerning our approach to the study of people’s perception of aggression-related risks. First, we limited the aggression-related domain to instrumental aggression, in which harmful actions have a purpose, such as winning a game, or more broadly solving a problem. However, there is another type of aggression, hostile aggression, which includes harmful actions motivated by anger and aimed to harm someone (Wann, 2005). Future research should consider a fine-grained and comprehensive study of perceived aggression-related risks by tapping these two types of aggression. Second, aggression-related issues are not the unique risk typically associated with the practice of sports. Other negative outcomes have been identified, such as musculoskeletal injury and adverse cardiovascular events (American College of Sports Medicine, 2014), or bullying, hazing, and harassment in youth sports, which carry risks of both physical and mental harm (McMullen, 2014). Finally, it should also be noted that the line between risks and benefits is not always clear. For example, though weight loss is typically seen as major benefit of PA in general (King et al., 2014), for underweight people or even athletes practicing weight-class sports, weight loss may be seen as a risk (Turocy et al., 2011). Overall, the benefits and aggression-related risks examined in this paper provide a snapshot that we hope will contribute to the understanding of the bigger picture in sports’ perceptions, clearly requiring further research attention.

## Conclusion

This work integrates a larger set of studies aimed to examine perceived benefits and aggression-related risks in sports. Findings provided preliminary validity and reliability evidence on the PBAR scale. Though requiring further testing with different populations and sports, this seems a promising tool to examine and compare people’s perceptions about different sports. Such examination is critical because more than the real benefits of a sport, it is the perceived outcomes that will influence individuals’ intention to engage in it. Information on people’s beliefs about sports outcomes can guide the design of strategies to maximize participation in sports that bring widespread benefits, such as football and karate.

## Data Availability Statement

The raw data supporting the conclusions of this article will be made available by the authors, without undue reservation.

## Ethics Statement

The studies involving human participants were reviewed and approved by the University of Porto. The patients/participants provided their written informed consent to participate in this study.

## Author Contributions

TL designed the study (including the PBAR scale), oversaw the data collection and coding, analyzed and interpreted the data, and wrote the first version of the manuscript. ST contributed to the design of the study (including the PBAR scale) and reviewed the manuscript. Both authors contributed to the article and approved the submitted version.

## Conflict of Interest

The authors declare that the research was conducted in the absence of any commercial or financial relationships that could be construed as a potential conflict of interest.
